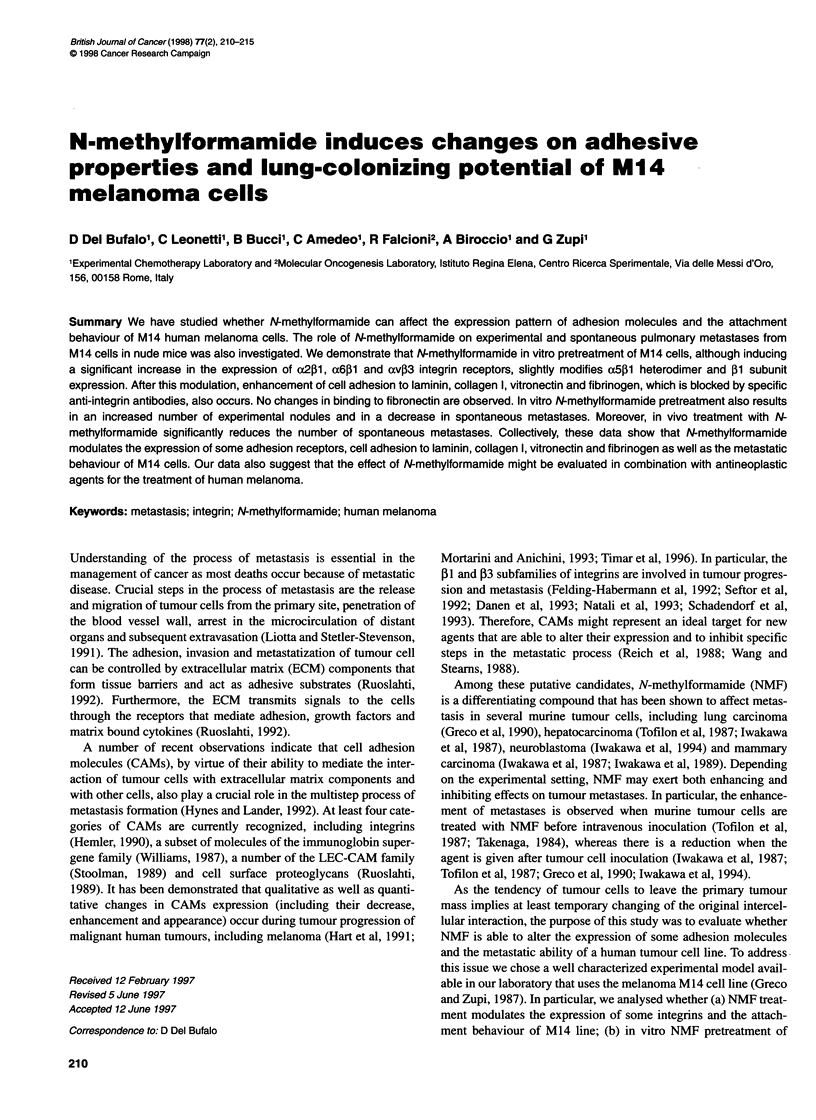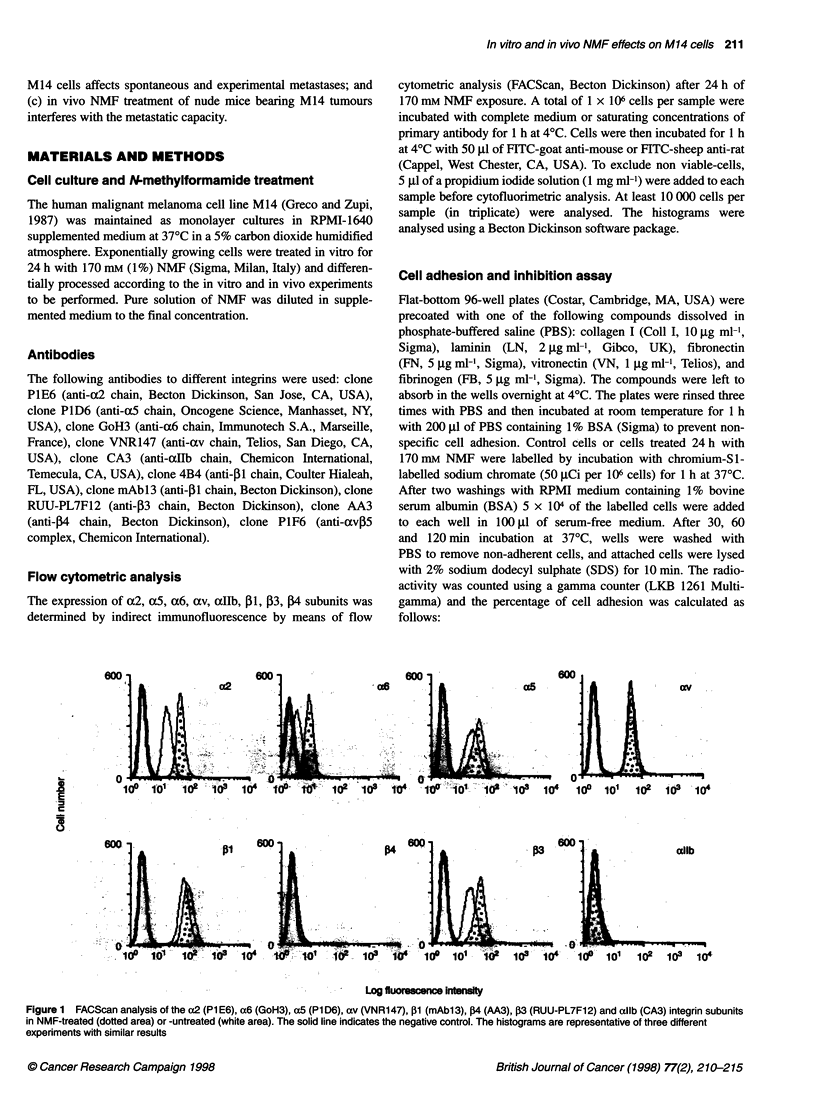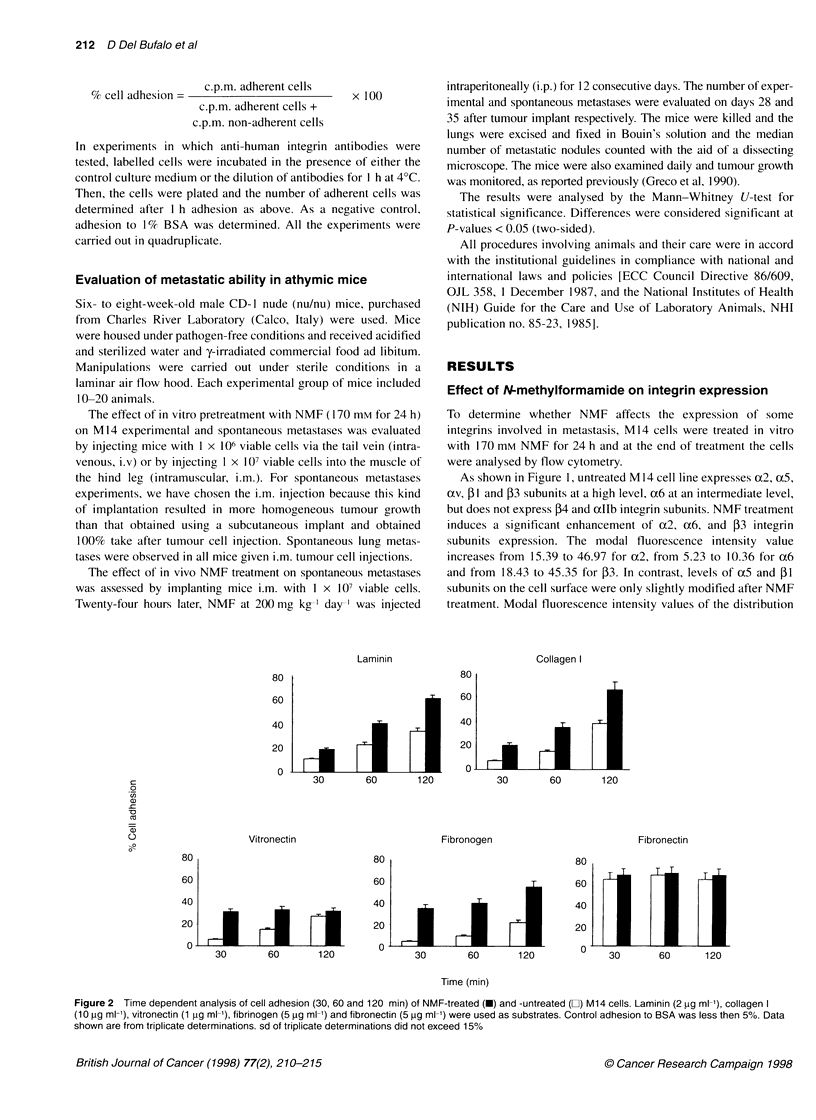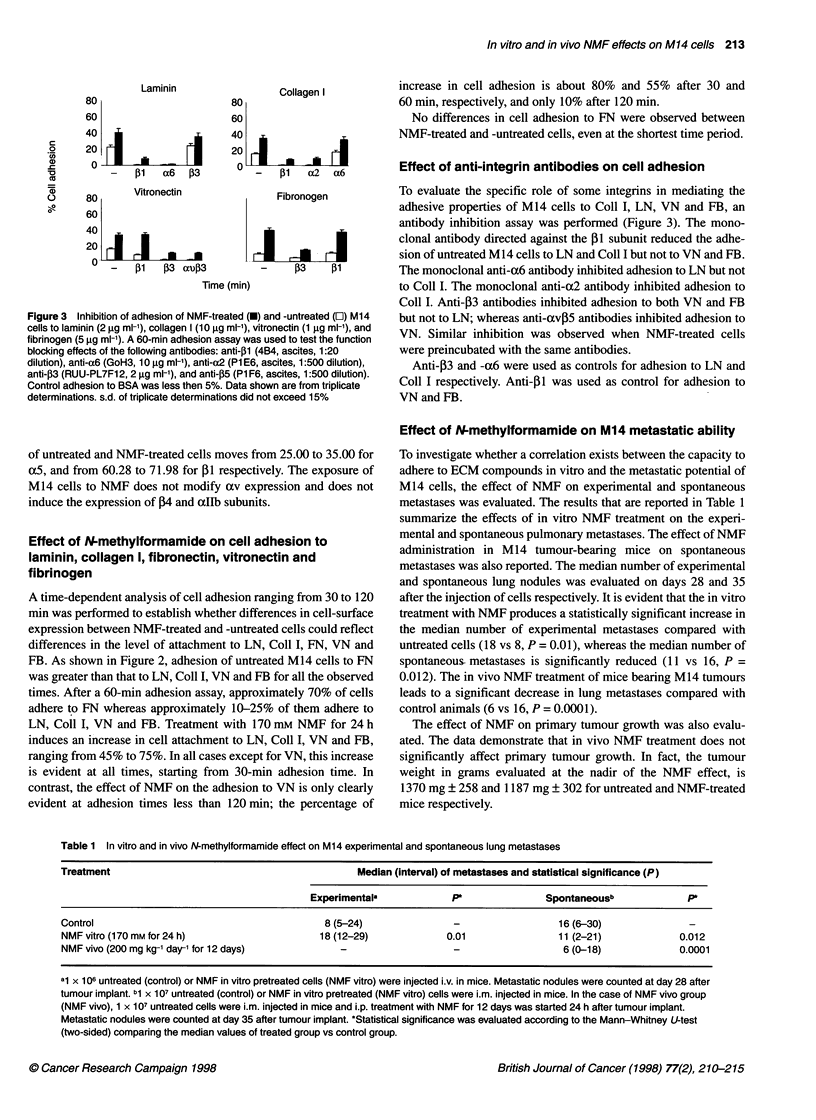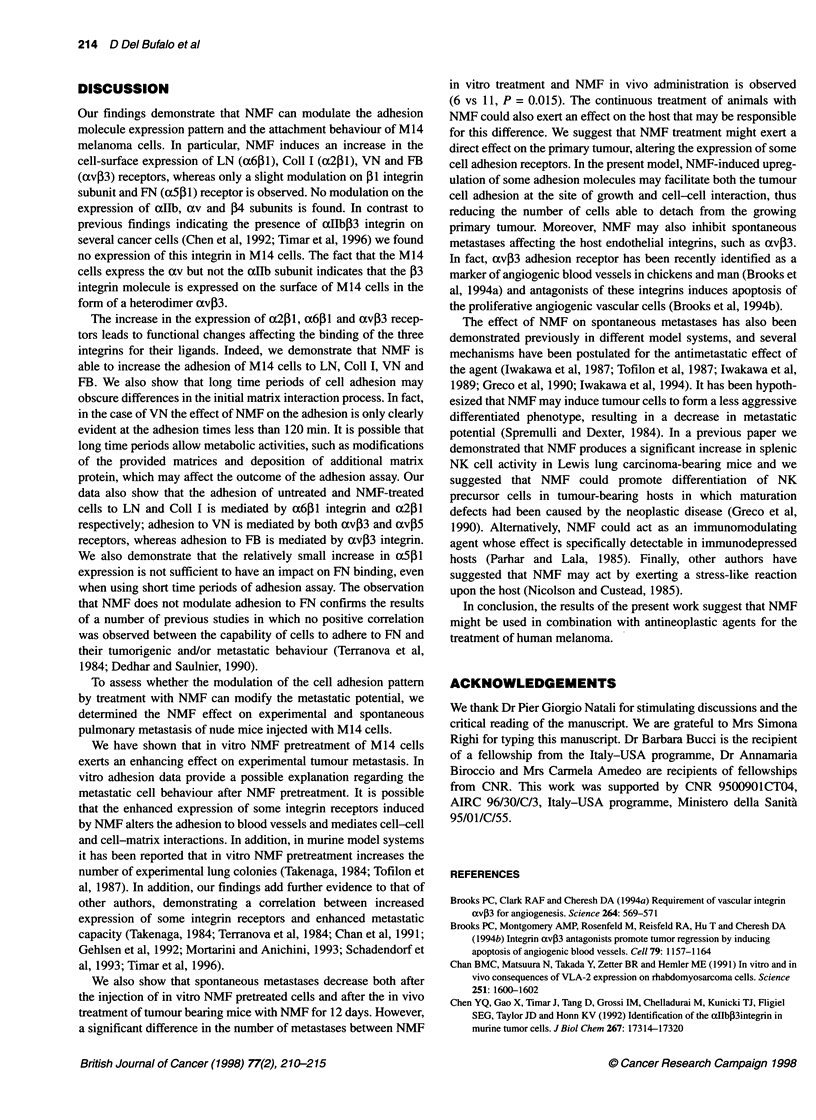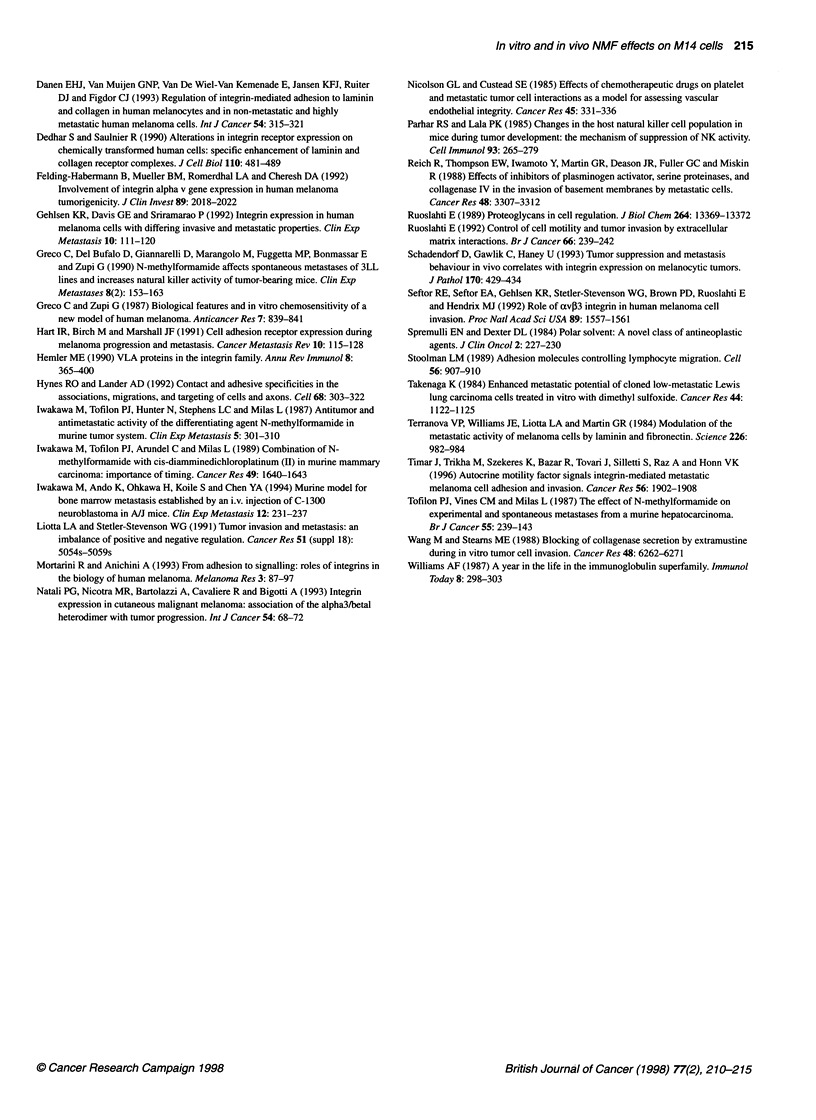# N-methylformamide induces changes on adhesive properties and lung-colonizing potential of M14 melanoma cells.

**DOI:** 10.1038/bjc.1998.35

**Published:** 1998

**Authors:** D. Del Bufalo, C. Leonetti, B. Bucci, C. Amedeo, R. Falcioni, A. Biroccio, G. Zupi

**Affiliations:** Experimental Chemotherapy Laboratory, Istituto Regina Elena, Centro Ricerca Sperimentale, Rome, Italy.

## Abstract

We have studied whether N-methylformamide can affect the expression pattern of adhesion molecules and the attachment behaviour of M14 human melanoma cells. The role of N-methylformamide on experimental and spontaneous pulmonary metastases from M14 cells in nude mice was also investigated. We demonstrate that N-methylformamide in vitro pretreatment of M14 cells, although inducing a significant increase in the expression of alpha2beta1, alpha6beta1 and alpha(v)beta3 integrin receptors, slightly modifies alpha5beta1 heterodimer and beta1 subunit expression. After this modulation, enhancement of cell adhesion to laminin, collagen I, vitronectin and fibrinogen, which is blocked by specific anti-integrin antibodies, also occurs. No changes in binding to fibronectin are observed. In vitro N-methylformamide pretreatment also results in an increased number of experimental nodules and in a decrease in spontaneous metastases. Moreover, in vivo treatment with N-methylformamide significantly reduces the number of spontaneous metastases. Collectively, these data show that N-methylformamide modulates the expression of some adhesion receptors, cell adhesion to laminin, collagen I, vitronectin and fibrinogen as well as the metastatic behaviour of M14 cells. Our data also suggest that the effect of N-methylformamide might be evaluated in combination with antineoplastic agents for the treatment of human melanoma.


British Journal of Cancer (1998) 77(2), 210–215
© 1998 Cancer Research Campaign

### N-methylformamide induces changes on adhesive properties and lung-colonizing potential of M14 melanoma cells

D Del Bufalo[1], C Leonetti[1], B Bucci[1], C Amedeo[1], R Falcioni[2], A Biroccio[1] and G Zupi[1]

[1]Experimental Chemotherapy Laboratory and [2]Molecular Oncogenesis Laboratory, Istituto Regina Elena, Centro Ricerca Sperimentale, Via delle Messi d'Oro, 156, 00158 Rome, Italy

**Summary** We have studied whether *N*-methylformamide can affect the expression pattern of adhesion molecules and the attachment behaviour of M14 human melanoma cells. The role of *N*-methylformamide on experimental and spontaneous pulmonary metastases from M14 cells in nude mice was also investigated. We demonstrate that *N*-methylformamide in vitro pretreatment of M14 cells, although inducing a significant increase in the expression of α2β1, α6β1 and αvβ3 integrin receptors, slightly modifies α5β1 heterodimer and β1 subunit expression. After this modulation, enhancement of cell adhesion to laminin, collagen I, vitronectin and fibrinogen, which is blocked by specific anti-integrin antibodies, also occurs. No changes in binding to fibronectin are observed. In vitro *N*-methylformamide pretreatment also results in an increased number of experimental nodules and in a decrease in spontaneous metastases. Moreover, in vivo treatment with *N*-methylformamide significantly reduces the number of spontaneous metastases. Collectively, these data show that *N*-methylformamide modulates the expression of some adhesion receptors, cell adhesion to laminin, collagen I, vitronectin and fibrinogen as well as the metastatic behaviour of M14 cells. Our data also suggest that the effect of *N*-methylformamide might be evaluated in combination with antineoplastic agents for the treatment of human melanoma.

**Keywords:** metastasis; integrin; *N*-methylformamide; human melanoma

Understanding of the process of metastasis is essential in the management of cancer as most deaths occur because of metastatic disease. Crucial steps in the process of metastasis are the release and migration of tumour cells from the primary site, penetration of the blood vessel wall, arrest in the microcirculation of distant organs and subsequent extravasation (Liotta and Stetler-Stevenson, 1991). The adhesion, invasion and metastatization of tumour cell can be controlled by extracellular matrix (ECM) components that form tissue barriers and act as adhesive substrates (Ruoslahti, 1992). Furthermore, the ECM transmits signals to the cells through the receptors that mediate adhesion, growth factors and matrix bound cytokines (Ruoslahti, 1992).

A number of recent observations indicate that cell adhesion molecules (CAMs), by virtue of their ability to mediate the interaction of tumour cells with extracellular matrix components and with other cells, also play a crucial role in the multistep process of metastasis formation (Hynes and Lander, 1992). At least four categories of CAMs are currently recognized, including integrins (Hemler, 1990), a subset of molecules of the immunoglobin supergene family (Williams, 1987), a number of the LEC-CAM family (Stoolman, 1989) and cell surface proteoglycans (Ruoslahti, 1989). It has been demonstrated that qualitative as well as quantitative changes in CAMs expression (including their decrease, enhancement and appearance) occur during tumour progression of malignant human tumours, including melanoma (Hart et al, 1991; Mortarini and Anichini, 1993; Timar et al, 1996). In particular, the β1 and β3 subfamilies of integrins are involved in tumour progression and metastasis (Felding-Habermann et al, 1992; Seftor et al, 1992; Danen et al, 1993; Natali et al, 1993; Schadendorf et al, 1993). Therefore, CAMs might represent an ideal target for new agents that are able to alter their expression and to inhibit specific steps in the metastatic process (Reich et al, 1988; Wang and Stearns, 1988).

Among these putative candidates, *N*-methylformamide (NMF) is a differentiating compound that has been shown to affect metastasis in several murine tumour cells, including lung carcinoma (Greco et al, 1990), hepatocarcinoma (Tofilon et al, 1987; Iwakawa et al, 1987), neuroblastoma (Iwakawa et al, 1994) and mammary carcinoma (Iwakawa et al, 1987; Iwakawa et al, 1989). Depending on the experimental setting, NMF may exert both enhancing and inhibiting effects on tumour metastases. In particular, the enhancement of metastases is observed when murine tumour cells are treated with NMF before intravenous inoculation (Tofilon et al, 1987; Takenaga, 1984), whereas there is a reduction when the agent is given after tumour cell inoculation (Iwakawa et al, 1987; Tofilon et al, 1987; Greco et al, 1990; Iwakawa et al, 1994).

As the tendency of tumour cells to leave the primary tumour mass implies at least temporary changing of the original intercellular interaction, the purpose of this study was to evaluate whether NMF is able to alter the expression of some adhesion molecules and the metastatic ability of a human tumour cell line. To address this issue we chose a well characterized experimental model available in our laboratory that uses the melanoma M14 cell line (Greco and Zupi, 1987). In particular, we analysed whether (a) NMF treatment modulates the expression of some integrins and the attachment behaviour of M14 line; (b) in vitro NMF pretreatment of

*Received 12 February 1997*
*Revised 5 June 1997*
*Accepted 12 June 1997*

*Correspondence to:* D Del Bufalo

M14 cells affects spontaneous and experimental metastases; and (c) in vivo NMF treatment of nude mice bearing M14 tumours interferes with the metastatic capacity.

### MATERIALS AND METHODS

### Cell culture and *N*-methylformamide treatment

The human malignant melanoma cell line M14 (Greco and Zupi, 1987) was maintained as monolayer cultures in RPMI-1640 supplemented medium at 37°C in a 5% carbon dioxide humidified atmosphere. Exponentially growing cells were treated in vitro for 24 h with 170 mM (1%) NMF (Sigma, Milan, Italy) and differentially processed according to the in vitro and in vivo experiments to be performed. Pure solution of NMF was diluted in supplemented medium to the final concentration.

### Antibodies

The following antibodies to different integrins were used: clone P1E6 (anti-α2 chain, Becton Dickinson, San Jose, CA, USA), clone P1D6 (anti-α5 chain, Oncogene Science, Manhasset, NY, USA), clone GoH3 (anti-α6 chain, Immunotech S.A., Marseille, France), clone VNR147 (anti-αv chain, Telios, San Diego, CA, USA), clone CA3 (anti-αIIb chain, Chemicon International, Temecula, CA, USA), clone 4B4 (anti-β1 chain, Coulter Hialeah, FL, USA), clone mAb13 (anti-β1 chain, Becton Dickinson), clone RUU-PL7F12 (anti-β3 chain, Becton Dickinson), clone AA3 (anti-β4 chain, Becton Dickinson), clone P1F6 (anti-αvβ5 complex, Chemicon International).

### Flow cytometric analysis

The expression of α2, α5, α6, αv, αIIb, β1, β3, β4 subunits was determined by indirect immunofluorescence by means of flow cytometric analysis (FACScan, Becton Dickinson) after 24 h of 170 mM NMF exposure. A total of $1 \times 10^6$ cells per sample were incubated with complete medium or saturating concentrations of primary antibody for 1 h at 4°C. Cells were then incubated for 1 h at 4°C with 50 μl of FITC-goat anti-mouse or FITC-sheep anti-rat (Cappel, West Chester, CA, USA). To exclude non viable-cells, 5 μl of a propidium iodide solution (1 mg $ml^{-1}$) were added to each sample before cytofluorimetric analysis. At least 10 000 cells per sample (in triplicate) were analysed. The histograms were analysed using a Becton Dickinson software package.

### Cell adhesion and inhibition assay

Flat-bottom 96-well plates (Costar, Cambridge, MA, USA) were precoated with one of the following compounds dissolved in phosphate-buffered saline (PBS): collagen I (Coll I, 10 μg $ml^{-1}$, Sigma), laminin (LN, 2 μg $ml^{-1}$, Gibco, UK), fibronectin (FN, 5 μg $ml^{-1}$, Sigma), vitronectin (VN, 1 μg $ml^{-1}$, Telios), and fibrinogen (FB, 5 μg $ml^{-1}$, Sigma). The compounds were left to absorb in the wells overnight at 4°C. The plates were rinsed three times with PBS and then incubated at room temperature for 1 h with 200 μl of PBS containing 1% BSA (Sigma) to prevent non-specific cell adhesion. Control cells or cells treated 24 h with 170 mM NMF were labelled by incubation with chromium-51-labelled sodium chromate (50 μCi per $10^6$ cells) for 1 h at 37°C. After two washings with RPMI medium containing 1% bovine serum albumin (BSA) $5 \times 10^4$ of the labelled cells were added to each well in 100 μl of serum-free medium. After 30, 60 and 120 min incubation at 37°C, wells were washed with PBS to remove non-adherent cells, and attached cells were lysed with 2% sodium dodecyl sulphate (SDS) for 10 min. The radioactivity was counted using a gamma counter (LKB 1261 Multigamma) and the percentage of cell adhesion was calculated as follows:

**Figure 1** FACScan analysis of the α2 (P1E6), α6 (GoH3), α5 (P1D6), αv (VNR147), β1 (mAb13), β4 (AA3), β3 (RUU-PL7F12) and αIIb (CA3) integrin subunits in NMF-treated (dotted area) or -untreated (white area). The solid line indicates the negative control. The histograms are representative of three different experiments with similar results

© Cancer Research Campaign 1998

$$\% \text{ cell adhesion} = \frac{\text{c.p.m. adherent cells}}{\text{c.p.m. adherent cells} + \text{c.p.m. non-adherent cells}} \times 100$$

In experiments in which anti-human integrin antibodies were tested, labelled cells were incubated in the presence of either the control culture medium or the dilution of antibodies for 1 h at 4°C. Then, the cells were plated and the number of adherent cells was determined after 1 h adhesion as above. As a negative control, adhesion to 1% BSA was determined. All the experiments were carried out in quadruplicate.

### Evaluation of metastatic ability in athymic mice

Six- to eight-week-old male CD-1 nude (nu/nu) mice, purchased from Charles River Laboratory (Calco, Italy) were used. Mice were housed under pathogen-free conditions and received acidified and sterilized water and γ-irradiated commercial food ad libitum. Manipulations were carried out under sterile conditions in a laminar air flow hood. Each experimental group of mice included 10–20 animals.

The effect of in vitro pretreatment with NMF (170 mM for 24 h) on M14 experimental and spontaneous metastases was evaluated by injecting mice with $1 \times 10^6$ viable cells via the tail vein (intravenous, i.v) or by injecting $1 \times 10^7$ viable cells into the muscle of the hind leg (intramuscular, i.m.). For spontaneous metastases experiments, we have chosen the i.m. injection because this kind of implantation resulted in more homogeneous tumour growth than that obtained using a subcutaneous implant and obtained 100% take after tumour cell injection. Spontaneous lung metastases were observed in all mice given i.m. tumour cell injections.

The effect of in vivo NMF treatment on spontaneous metastases was assessed by implanting mice i.m. with $1 \times 10^7$ viable cells. Twenty-four hours later, NMF at 200 mg $kg^{-1}$ $day^{-1}$ was injected intraperitoneally (i.p.) for 12 consecutive days. The number of experimental and spontaneous metastases were evaluated on days 28 and 35 after tumour implant respectively. The mice were killed and the lungs were excised and fixed in Bouin's solution and the median number of metastatic nodules counted with the aid of a dissecting microscope. The mice were also examined daily and tumour growth was monitored, as reported previously (Greco et al, 1990).

The results were analysed by the Mann–Whitney *U*-test for statistical significance. Differences were considered significant at *P*-values < 0.05 (two-sided).

All procedures involving animals and their care were in accord with the institutional guidelines in compliance with national and international laws and policies [ECC Council Directive 86/609, OJL 358, 1 December 1987, and the National Institutes of Health (NIH) Guide for the Care and Use of Laboratory Animals, NHI publication no. 85-23, 1985].

### RESULTS

### Effect of *N*-methylformamide on integrin expression

To determine whether NMF affects the expression of some integrins involved in metastasis, M14 cells were treated in vitro with 170 mM NMF for 24 h and at the end of treatment the cells were analysed by flow cytometry.

As shown in Figure 1, untreated M14 cell line expresses α2, α5, αv, β1 and β3 subunits at a high level, α6 at an intermediate level, but does not express β4 and αIIb integrin subunits. NMF treatment induces a significant enhancement of α2, α6, and β3 integrin subunits expression. The modal fluorescence intensity value increases from 15.39 to 46.97 for α2, from 5.23 to 10.36 for α6 and from 18.43 to 45.35 for β3. In contrast, levels of α5 and β1 subunits on the cell surface were only slightly modified after NMF treatment. Modal fluorescence intensity values of the distribution

**Figure 2** Time dependent analysis of cell adhesion (30, 60 and 120 min) of NMF-treated (■) and -untreated (□) M14 cells. Laminin (2 μg $ml^{-1}$), collagen I (10 μg $ml^{-1}$), vitronectin (1 μg $ml^{-1}$), fibrinogen (5 μg $ml^{-1}$) and fibronectin (5 μg $ml^{-1}$) were used as substrates. Control adhesion to BSA was less then 5%. Data shown are from triplicate determinations. sd of triplicate determinations did not exceed 15%

© Cancer Research Campaign 1998

**Figure 3** Inhibition of adhesion of NMF-treated (■) and -untreated (□) M14 cells to laminin (2 μg ml$^{-1}$), collagen I (10 μg ml$^{-1}$), vitronectin (1 μg ml$^{-1}$), and fibrinogen (5 μg ml$^{-1}$). A 60-min adhesion assay was used to test the function blocking effects of the following antibodies: anti-β1 (4B4, ascites, 1:20 dilution), anti-α6 (GoH3, 10 μg ml$^{-1}$), anti-α2 (P1E6, ascites, 1:500 dilution), anti-β3 (RUU-PL7F12, 2 μg ml$^{-1}$), and anti-β5 (P1F6, ascites, 1:500 dilution). Control adhesion to BSA was less then 5%. Data shown are from triplicate determinations. s.d. of triplicate determinations did not exceed 15%

of untreated and NMF-treated cells moves from 25.00 to 35.00 for α5, and from 60.28 to 71.98 for β1 respectively. The exposure of M14 cells to NMF does not modify αv expression and does not induce the expression of β4 and αIIb subunits.

### Effect of *N*-methylformamide on cell adhesion to laminin, collagen I, fibronectin, vitronectin and fibrinogen

A time-dependent analysis of cell adhesion ranging from 30 to 120 min was performed to establish whether differences in cell-surface expression between NMF-treated and -untreated cells could reflect differences in the level of attachment to LN, Coll I, FN, VN and FB. As shown in Figure 2, adhesion of untreated M14 cells to FN was greater than that to LN, Coll I, VN and FB for all the observed times. After a 60-min adhesion assay, approximately 70% of cells adhere to FN whereas approximately 10–25% of them adhere to LN, Coll I, VN and FB. Treatment with 170 mM NMF for 24 h induces an increase in cell attachment to LN, Coll I, VN and FB, ranging from 45% to 75%. In all cases except for VN, this increase is evident at all times, starting from 30-min adhesion time. In contrast, the effect of NMF on the adhesion to VN is only clearly evident at adhesion times less than 120 min; the percentage of increase in cell adhesion is about 80% and 55% after 30 and 60 min, respectively, and only 10% after 120 min.

No differences in cell adhesion to FN were observed between NMF-treated and -untreated cells, even at the shortest time period.

### Effect of anti-integrin antibodies on cell adhesion

To evaluate the specific role of some integrins in mediating the adhesive properties of M14 cells to Coll I, LN, VN and FB, an antibody inhibition assay was performed (Figure 3). The monoclonal antibody directed against the β1 subunit reduced the adhesion of untreated M14 cells to LN and Coll I but not to VN and FB. The monoclonal anti-α6 antibody inhibited adhesion to LN but not to Coll I. The monoclonal anti-α2 antibody inhibited adhesion to Coll I. Anti-β3 antibodies inhibited adhesion to both VN and FB but not to LN; whereas anti-αvβ5 antibodies inhibited adhesion to VN. Similar inhibition was observed when NMF-treated cells were preincubated with the same antibodies.

Anti-β3 and -α6 were used as controls for adhesion to LN and Coll I respectively. Anti-β1 was used as control for adhesion to VN and FB.

### Effect of *N*-methylformamide on M14 metastatic ability

To investigate whether a correlation exists between the capacity to adhere to ECM compounds in vitro and the metastatic potential of M14 cells, the effect of NMF on experimental and spontaneous metastases was evaluated. The results that are reported in Table 1 summarize the effects of in vitro NMF treatment on the experimental and spontaneous pulmonary metastases. The effect of NMF administration in M14 tumour-bearing mice on spontaneous metastases was also reported. The median number of experimental and spontaneous lung nodules was evaluated on days 28 and 35 after the injection of cells respectively. It is evident that the in vitro treatment with NMF produces a statistically significant increase in the median number of experimental metastases compared with untreated cells (18 vs 8, $P = 0.01$), whereas the median number of spontaneous metastases is significantly reduced (11 vs 16, $P = 0.012$). The in vivo NMF treatment of mice bearing M14 tumours leads to a significant decrease in lung metastases compared with control animals (6 vs 16, $P = 0.0001$).

The effect of NMF on primary tumour growth was also evaluated. The data demonstrate that in vivo NMF treatment does not significantly affect primary tumour growth. In fact, the tumour weight in grams evaluated at the nadir of the NMF effect, is 1370 mg ± 258 and 1187 mg ± 302 for untreated and NMF-treated mice respectively.

**Table 1** In vitro and in vivo *N*-methylformamide effect on M14 experimental and spontaneous lung metastases

| Treatment | Median (interval) of metastases and statistical significance (*P*) | | | |
|---|---|---|---|---|
| | Experimental[a] | *P*[*] | Spontaneous[b] | *P*[*] |
| Control | 8 (5–24) | – | 16 (6–30) | – |
| NMF vitro (170 mM for 24 h) | 18 (12–29) | 0.01 | 11 (2–21) | 0.012 |
| NMF vivo (200 mg kg$^{-1}$ day$^{-1}$ for 12 days) | – | – | 6 (0–18) | 0.0001 |

[a]1 × 10$^{6}$ untreated (control) or NMF in vitro pretreated cells (NMF vitro) were injected i.v. in mice. Metastatic nodules were counted at day 28 after tumour implant. [b]1 × 10$^{7}$ untreated (control) or NMF in vitro pretreated (NMF vitro) cells were i.m. injected in mice. In the case of NMF vivo group (NMF vivo), 1 × 10$^{7}$ untreated cells were i.m. injected in mice and i.p. treatment with NMF for 12 days was started 24 h after tumour implant. Metastatic nodules were counted at day 35 after tumour implant. [*]Statistical significance was evaluated according to the Mann–Whitney *U*-test (two-sided) comparing the median values of treated group vs control group.

© Cancer Research Campaign 1998 

### DISCUSSION

Our findings demonstrate that NMF can modulate the adhesion molecule expression pattern and the attachment behaviour of M14 melanoma cells. In particular, NMF induces an increase in the cell-surface expression of LN (α6β1), Coll I (α2β1), VN and FB (αvβ3) receptors, whereas only a slight modulation on β1 integrin subunit and FN (α5β1) receptor is observed. No modulation on the expression of αIIb, αv and β4 subunits is found. In contrast to previous findings indicating the presence of αIIbβ3 integrin on several cancer cells (Chen et al, 1992; Timar et al, 1996) we found no expression of this integrin in M14 cells. The fact that the M14 cells express the αv but not the αIIb subunit indicates that the β3 integrin molecule is expressed on the surface of M14 cells in the form of a heterodimer αvβ3.

The increase in the expression of α2β1, α6β1 and αvβ3 receptors leads to functional changes affecting the binding of the three integrins for their ligands. Indeed, we demonstrate that NMF is able to increase the adhesion of M14 cells to LN, Coll I, VN and FB. We also show that long time periods of cell adhesion may obscure differences in the initial matrix interaction process. In fact, in the case of VN the effect of NMF on the adhesion is only clearly evident at the adhesion times less than 120 min. It is possible that long time periods allow metabolic activities, such as modifications of the provided matrices and deposition of additional matrix protein, which may affect the outcome of the adhesion assay. Our data also show that the adhesion of untreated and NMF-treated cells to LN and Coll I is mediated by α6β1 integrin and α2β1 respectively; adhesion to VN is mediated by both αvβ3 and αvβ5 receptors, whereas adhesion to FB is mediated by αvβ3 integrin. We also demonstrate that the relatively small increase in α5β1 expression is not sufficient to have an impact on FN binding, even when using short time periods of adhesion assay. The observation that NMF does not modulate adhesion to FN confirms the results of a number of previous studies in which no positive correlation was observed between the capability of cells to adhere to FN and their tumorigenic and/or metastatic behaviour (Terranova et al, 1984; Dedhar and Saulnier, 1990).

To assess whether the modulation of the cell adhesion pattern by treatment with NMF can modify the metastatic potential, we determined the NMF effect on experimental and spontaneous pulmonary metastasis of nude mice injected with M14 cells.

We have shown that in vitro NMF pretreatment of M14 cells exerts an enhancing effect on experimental tumour metastasis. In vitro adhesion data provide a possible explanation regarding the metastatic cell behaviour after NMF pretreatment. It is possible that the enhanced expression of some integrin receptors induced by NMF alters the adhesion to blood vessels and mediates cell–cell and cell–matrix interactions. In addition, in murine model systems it has been reported that in vitro NMF pretreatment increases the number of experimental lung colonies (Takenaga, 1984; Tofilon et al, 1987). In addition, our findings add further evidence to that of other authors, demonstrating a correlation between increased expression of some integrin receptors and enhanced metastatic capacity (Takenaga, 1984; Terranova et al, 1984; Chan et al, 1991; Gehlsen et al, 1992; Mortarini and Anichini, 1993; Schadendorf et al, 1993; Timar et al, 1996).

We also show that spontaneous metastases decrease both after the injection of in vitro NMF pretreated cells and after the in vivo treatment of tumour bearing mice with NMF for 12 days. However, a significant difference in the number of metastases between NMF in vitro treatment and NMF in vivo administration is observed (6 vs 11, $P = 0.015$). The continuous treatment of animals with NMF could also exert an effect on the host that may be responsible for this difference. We suggest that NMF treatment might exert a direct effect on the primary tumour, altering the expression of some cell adhesion receptors. In the present model, NMF-induced upregulation of some adhesion molecules may facilitate both the tumour cell adhesion at the site of growth and cell–cell interaction, thus reducing the number of cells able to detach from the growing primary tumour. Moreover, NMF may also inhibit spontaneous metastases affecting the host endothelial integrins, such as αvβ3. In fact, αvβ3 adhesion receptor has been recently identified as a marker of angiogenic blood vessels in chickens and man (Brooks et al, 1994a) and antagonists of these integrins induces apoptosis of the proliferative angiogenic vascular cells (Brooks et al, 1994b).

The effect of NMF on spontaneous metastases has also been demonstrated previously in different model systems, and several mechanisms have been postulated for the antimetastatic effect of the agent (Iwakawa et al, 1987; Tofilon et al, 1987; Iwakawa et al, 1989; Greco et al, 1990; Iwakawa et al, 1994). It has been hypothesized that NMF may induce tumour cells to form a less aggressive differentiated phenotype, resulting in a decrease in metastatic potential (Spremulli and Dexter, 1984). In a previous paper we demonstrated that NMF produces a significant increase in splenic NK cell activity in Lewis lung carcinoma-bearing mice and we suggested that NMF could promote differentiation of NK precursor cells in tumour-bearing hosts in which maturation defects had been caused by the neoplastic disease (Greco et al, 1990). Alternatively, NMF could act as an immunomodulating agent whose effect is specifically detectable in immunodepressed hosts (Parhar and Lala, 1985). Finally, other authors have suggested that NMF may act by exerting a stress-like reaction upon the host (Nicolson and Custead, 1985).

In conclusion, the results of the present work suggest that NMF might be used in combination with antineoplastic agents for the treatment of human melanoma.

### ACKNOWLEDGEMENTS

We thank Dr Pier Giorgio Natali for stimulating discussions and the critical reading of the manuscript. We are grateful to Mrs Simona Righi for typing this manuscript. Dr Barbara Bucci is the recipient of a fellowship from the Italy–USA programme, Dr Annamaria Biroccio and Mrs Carmela Amedeo are recipients of fellowships from CNR. This work was supported by CNR 9500901CT04, AIRC 96/30/C/3, Italy–USA programme, Ministero della Sanità 95/01/C/55.

### REFERENCES

Brooks PC, Clark RAF and Cheresh DA (1994*a*) Requirement of vascular integrin αvβ3 for angiogenesis. *Science* **264**: 569–571

Brooks PC, Montgomery AMP, Rosenfeld M, Reisfeld RA, Hu T and Cheresh DA (1994*b*) Integrin αvβ3 antagonists promote tumor regression by inducing apoptosis of angiogenic blood vessels. *Cell* **79**: 1157–1164

Chan BMC, Matsuura N, Takada Y, Zetter BR and Hemler ME (1991) In vitro and in vivo consequences of VLA-2 expression on rhabdomyosarcoma cells. *Science* **251**: 1600–1602

Chen YQ, Gao X, Timar J, Tang D, Grossi IM, Chelladurai M, Kunicki TJ, Fligiel SEG, Taylor JD and Honn KV (1992) Identification of the αIIbβ3integrin in murine tumor cells. *J Biol Chem* **267**: 17314–17320

 © Cancer Research Campaign 1998

Danen EHJ, Van Muijen GNP, Van De Wiel-Van Kemenade E, Jansen KFJ, Ruiter DJ and Figdor CJ (1993) Regulation of integrin-mediated adhesion to laminin and collagen in human melanocytes and in non-metastatic and highly metastatic human melanoma cells. *Int J Cancer* **54**: 315–321

Dedhar S and Saulnier R (1990) Alterations in integrin receptor expression on chemically transformed human cells: specific enhancement of laminin and collagen receptor complexes. *J Cell Biol* **110**: 481–489

Felding-Habermann B, Mueller BM, Romerdhal LA and Cheresh DA (1992) Involvement of integrin alpha v gene expression in human melanoma tumorigenicity. *J Clin Invest* **89**: 2018–2022

Gehlsen KR, Davis GE and Sriramarao P (1992) Integrin expression in human melanoma cells with differing invasive and metastatic properties. *Clin Exp Metastasis* **10**: 111–120

Greco C, Del Bufalo D, Giannarelli D, Marangolo M, Fuggetta MP, Bonmassar E and Zupi G (1990) N-methylformamide affects spontaneous metastases of 3LL lines and increases natural killer activity of tumor-bearing mice. *Clin Exp Metastases* **8**(2): 153–163

Greco C and Zupi G (1987) Biological features and in vitro chemosensitivity of a new model of human melanoma. *Anticancer Res* **7**: 839–841

Hart IR, Birch M and Marshall JF (1991) Cell adhesion receptor expression during melanoma progression and metastasis. *Cancer Metastasis Rev* **10**: 115–128

Hemler ME (1990) VLA proteins in the integrin family. *Annu Rev Immunol* **8**: 365–400

Hynes RO and Lander AD (1992) Contact and adhesive specificities in the associations, migrations, and targeting of cells and axons. *Cell* **68**: 303–322

Iwakawa M, Tofilon PJ, Hunter N, Stephens LC and Milas L (1987) Antitumor and antimetastatic activity of the differentiating agent N-methylformamide in murine tumor system. *Clin Exp Metastasis* **5**: 301–310

Iwakawa M, Tofilon PJ, Arundel C and Milas L (1989) Combination of N-methylformamide with cis-diamminedichloroplatinum (II) in murine mammary carcinoma: importance of timing. *Cancer Res* **49**: 1640–1643

Iwakawa M, Ando K, Ohkawa H, Koile S and Chen YA (1994) Murine model for bone marrow metastasis established by an i.v. injection of C-1300 neuroblastoma in A/J mice. *Clin Exp Metastasis* **12**: 231–237

Liotta LA and Stetler-Stevenson WG (1991) Tumor invasion and metastasis: an imbalance of positive and negative regulation. *Cancer Res* **51** (suppl 18): 5054s–5059s

Mortarini R and Anichini A (1993) From adhesion to signalling: roles of integrins in the biology of human melanoma. *Melanoma Res* **3**: 87–97

Natali PG, Nicotra MR, Bartolazzi A, Cavaliere R and Bigotti A (1993) Integrin expression in cutaneous malignant melanoma: association of the alpha3/betal heterodimer with tumor progression. *Int J Cancer* **54**: 68–72

Nicolson GL and Custead SE (1985) Effects of chemotherapeutic drugs on platelet and metastatic tumor cell interactions as a model for assessing vascular endothelial integrity. *Cancer Res* **45**: 331–336

Parhar RS and Lala PK (1985) Changes in the host natural killer cell population in mice during tumor development: the mechanism of suppression of NK activity. *Cell Immunol* **93**: 265–279

Reich R, Thompson EW, Iwamoto Y, Martin GR, Deason JR, Fuller GC and Miskin R (1988) Effects of inhibitors of plasminogen activator, serine proteinases, and collagenase IV in the invasion of basement membranes by metastatic cells. *Cancer Res* **48**: 3307–3312

Ruoslahti E (1989) Proteoglycans in cell regulation. *J Biol Chem* **264**: 13369–13372

Ruoslahti E (1992) Control of cell motility and tumor invasion by extracellular matrix interactions. *Br J Cancer* **66**: 239–242

Schadendorf D, Gawlik C, Haney U (1993) Tumor suppression and metastasis behaviour in vivo correlates with integrin expression on melanocytic tumors. *J Pathol* **170**: 429–434

Seftor RE, Seftor EA, Gehlsen KR, Stetler-Stevenson WG, Brown PD, Ruoslahti E and Hendrix MJ (1992) Role of $\alpha v\beta 3$ integrin in human melanoma cell invasion. *Proc Natl Acad Sci USA* **89**: 1557–1561

Spremulli EN and Dexter DL (1984) Polar solvent: A novel class of antineoplastic agents. *J Clin Oncol* **2**: 227–230

Stoolman LM (1989) Adhesion molecules controlling lymphocyte migration. *Cell* **56**: 907–910

Takenaga K (1984) Enhanced metastatic potential of cloned low-metastatic Lewis lung carcinoma cells treated in vitro with dimethyl sulfoxide. *Cancer Res* **44**: 1122–1125

Terranova VP, Williams JE, Liotta LA and Martin GR (1984) Modulation of the metastatic activity of melanoma cells by laminin and fibronectin. *Science* **226**: 982–984

Timar J, Trikha M, Szekeres K, Bazar R, Tovari J, Silletti S, Raz A and Honn VK (1996) Autocrine motility factor signals integrin-mediated metastatic melanoma cell adhesion and invasion. *Cancer Res* **56**: 1902–1908

Tofilon PJ, Vines CM and Milas L (1987) The effect of N-methylformamide on experimental and spontaneous metastases from a murine hepatocarcinoma. *Br J Cancer* **55**: 239–143

Wang M and Stearns ME (1988) Blocking of collagenase secretion by extramustine during in vitro tumor cell invasion. *Cancer Res* **48**: 6262–6271

Williams AF (1987) A year in the life in the immunoglobulin superfamily. *Immunol Today* **8**: 298–303

© Cancer Research Campaign 1998